# Frequency-domain band-pass filtering enhanced pulsation method for pulmonary embolism diagnosis and risk stratification: a two-center retrospective study

**DOI:** 10.1186/s13054-026-06173-9

**Published:** 2026-07-10

**Authors:** Shaofei Xu, Jiazheng Li, Ziqi Li, Yuxuan Cai, Junlai Zhao, Rongrong Zhu, Maokun Li, Weiwei Wu

**Affiliations:** 1 Department of Vascular Surgery, Beijing Tsinghua Changgung Hospital, School of Clinical Medicine, Tsinghua Medicine, Tsinghua University, No. 168 Litang Road, Changping District, Beijing, 102218 China; 2 Department of Electronic Engineering, Tsinghua University, Beijing, 100084 China; 3 Department of Vascular Surgery, Chenzhou First People’s Hospital and the first Affiliated Hospital of Xiangnan University, Chenzhou, 423000 Hunan Province China

**Keywords:** Electrical impedance tomography, Pulmonary embolism, Frequency-domain band-pass filtering, Pulsatility method, Risk stratification, Embolus location

## Abstract

**Background:**

Pulmonary embolism (PE) remains a major cause of morbidity and mortality in critical care, yet traditional diagnostic methods face limitations, especially in critically ill patients. This study introduces a novel frequency-domain band-pass filtering enhanced electrical impedance tomography (EIT) pulsation method for rapid, non-invasive PE diagnosis and risk stratification.

**Methods:**

In a two-center retrospective study, 106 participants (53 PE patients, 53 healthy controls) were enrolled. A 16-electrode EIT system recorded pulmonary blood flow pulsation signals, and a heart rate-adaptive band-pass filter with its lower cutoff frequency set slightly below the estimated heart rate was applied to extract perfusion-related pulsatility while separating it from ventilation-related low-frequency components. Key parameters (Matching Index (MI), Dead space index (DI), Shunt Index (SI), Electrical Impedance VQ ratio(EIVQ)) were analyzed for PE diagnosis and risk stratification.

**Results:**

Compared with healthy controls, PE patients had significantly lower MI (*P* < 0.001) and higher DI (*P* < 0.001) and SI (*P* = 0.005). For risk stratification, intermediate-high-risk PE patients showed lower MI and higher DI than lower-risk patients, with the strongest differences versus low-risk patients (both adjusted *P* < 0.001).Among the EIT-derived parameters, the combined MI + DI+SI model achieved the highest AUC (0.820, 95% CI: 0.741–0.899), although the improvement over MI alone was marginal; it outperformed DI and SI. EIVQ showed no significant discriminative value.

**Conclusions:**

The enhanced EIT pulsatility method shows promise for non-invasive bedside assessment of PE-related perfusion abnormalities. MI, DI, and SI differentiated PE patients from healthy controls, while MI and DI were associated with risk stratification. These findings support the diagnostic and risk-stratification potential of EIT-derived pulsatility parameters and warrant further validation in consecutive patients with suspected PE.

**Supplementary Information:**

The online version contains supplementary material available at https://doi.org/10.1186/s13054-026-06173-9.

## Introduction

Pulmonary embolism (PE) is a life-threatening cardiovascular emergency and ranks as the third leading cause of cardiovascular mortality worldwide. The in-hospital mortality rate for untreated PE is 20–30%, with mortality rates exceeding 60% in critically ill intensive care unit (ICU) patients [1–3]. For ICU patients, prompt bedside assessment is crucial, as delayed diagnosis and intervention can significantly worsen outcomes.

Computed tomography pulmonary angiography (CTPA), the gold standard for PE diagnosis, has inherent limitations for critically ill patients. These include ionizing radiation exposure, the risk of contrast-induced nephropathy—particularly in patients with renal impairment—and the need for patient transfer, which may be unfeasible for hemodynamically unstable individuals [4–5]. D-dimer, a commonly used non-invasive screening tool, has poor specificity (40–60%) in elderly, post-surgical, and oncological patients, who are core ICU subgroups. This leads to overutilization of CTPA and unnecessary resource consumption [6]. Although clinical probability scores such as 4PEPS and YEARS have optimized CTPA use by reducing unnecessary scans [7–8], they lack direct diagnostic value and do not address the need for real-time bedside perfusion assessment.

Pulsation-based pulmonary perfusion assessment, particularly using electrical impedance tomography (EIT) in pulsation mode, has emerged as a promising non-invasive method for evaluating ventilation-perfusion (V/Q) matching, a key pathophysiological feature of PE [9]. Advances such as three-dimensional EIT and saline contrast-enhanced techniques have improved perfusion imaging. However, conventional EIT approaches still face limitations due to signal artifacts, including respiratory-induced impedance changes, patient movement, and conductivity drift [10–11]. Standard low-pass filtering can attenuate respiratory harmonics but may also distort the amplitude and timing of cardiac signals. Additionally, traditional saline-contrast EIT studies rely on rapid injection of a hypertonic saline bolus through a central venous catheter, typically during a breath-hold of 8–15 s, which restricts use in ICU patients who cannot tolerate apnea [12–13].

To overcome these limitations, we developed a heart rate–adaptive frequency-domain band-pass filtering method. This method uses a 0.8–2 Hz band-pass filter tuned to the cardiac cycle, preserving pulmonary blood flow signals while suppressing respiratory (0.2–0.33 Hz) and low-frequency motion (< 0.1 Hz) artifacts [14]. Compared with non-adaptive filtering strategies, the heart rate-adaptive approach aligns the retained frequency range with each patient’s cardiac rhythm, helping to preserve cardiac-synchronous pulsatility signals while reducing contamination from respiratory-related components [15].

In this two-center retrospective study, we assessed the diagnostic performance of this heart rate–adaptive, frequency-domain filtered EIT method in critically ill patients with PE. To our knowledge, this is the first focused evaluation of such an approach for PE diagnosis in the ICU, providing evidence for a rapid, non-invasive bedside tool that bridges the gap between emerging pulmonary perfusion imaging technologies and their clinical implementation.

## Materials and methods

### Study design and participants

This two-center, retrospective case-control study was conducted at Tsinghua Chang Gung Hospital in Beijing and Chenzhou First People’s Hospital in Hunan. The study was approved by the institutional review boards of both centers and conducted in accordance with the Declaration of Helsinki (2013 revision). Informed consent was obtained from all participants or their legally authorized representatives. PE patients confirmed by CTPA were enrolled. All patients were treatment-naïve with complete clinical data, including biomarkers for disease severity. Healthy controls were individuals without clinical suspicion of pulmonary embolism, with normal health-screening findings, and with no history of significant cardiopulmonary disease, recent surgery, trauma, infection, pregnancy, or lactation. They were not patients evaluated for suspected PE with negative CTPA findings. Exclusion criteria included severe pre-existing cardiopulmonary dysfunction unrelated to PE and incomplete clinical, EIT, or follow-up data. PE patients were independently evaluated by two senior radiologists and intensivists in a double-blind manner, with disagreements resolved by consensus. Embolus location was classified as central (main pulmonary artery or left/right main pulmonary arteries) or non-central (lobar, segmental, or subsegmental arteries). Key hemodynamic and laboratory parameters were used to assess disease severity and prognostic risk.

### Frequency-domain band-pass filtering enhanced pulsation method

Pulsation imaging was performed using a dedicated system (Model ET1000, Infivision Medical Imaging Technology Co, Ltd, Beijing, China) with a 16-electrode chest belt and an integrated signal processing workstation. Measurements were conducted at the bedside, lasting about 5 min. Participants were positioned supine, with mechanically ventilated patients maintained on their original ventilator settings. The electrode belt was placed at the 3rd–5th intercostal spaces using conductive paste, where the measurement plane is sensitive to impedance changes originating from the pulmonary vascular bed, and pulmonary blood flow signals were recorded at 20 Hz for 60 s. Raw signals were processed using Fast Fourier Transform and a heart rate-adaptive band-pass filtering procedure to isolate cardiac-related pulsations. Heart rate was estimated directly from the EIT pulsatility signal using sliding-window spectral analysis. A 15-second window with a 1-second step was used to calculate the spectrum of the signal within each window. The dominant respiratory component was first identified, and the respiratory frequency and its harmonics were removed from the spectrum. The largest remaining spectral peak within the predefined cardiac frequency range was then defined as the instantaneous heart-rate frequency. The center frequency of the band-pass filter was dynamically set to the estimated heart-rate frequency, with a bandwidth of ± 0.2 Hz. The final passband was constrained within the predefined cardiac frequency range of 0.8–2.0 Hz.Respiratory interference and low-frequency motion were attenuated using notch and sliding-average filters. The signals were reconstructed in MATLAB R2023b through differential processing and periodic superposition averaging. To reduce non-pulmonary cardiac artifacts, the filtered pulsatility signals were further analyzed within lung-region ROIs. Boundary-related signals and mediastinal components were excluded during image post-processing and ROI extraction, thereby emphasizing impedance changes distributed within the lung fields.Four quantitative indices were derived to evaluate ventilation–perfusion matching: the Matching Index (MI), Dead Space Index (DI), Shunt Index (SI), and Electrical Impedance V/Q ratio (EIVQ). These indices were calculated as follows.

1 $$\:\mathrm{MI}\:\mathrm{=}\text{}\frac{\sum_{{i}\mathrm{=1}}^{{N}}\mathrm{(}{P}\mathrm{(}{i}\mathrm{)}\times{V}\mathrm{(}{i}\mathrm{)}{\mathrm{)}}^{\mathrm{2}}}{\sum_{{i}\mathrm{=1}}^{{N}}{{P}}^{\mathrm{2}}\mathrm{(}{i}\mathrm{)}\times\sum_{{i}\mathrm{=1}}^{{N}}{{V}}^{\mathrm{2}}\mathrm{(}{i}\mathrm{)}}\text{}\times 100\%$$

2 $$\:\text{}{DI}\mathrm{=}\frac{\sum_{{i}\mathrm{=1}}^{{N}}{{V}}^{\mathrm{2}}\mathrm{(}{i}\mathrm{)}-\sum_{{i}\mathrm{=1}}^{{N}}\mathrm{(}{P}\mathrm{(}{i}\mathrm{)}\times{V}\mathrm{(}{i}\mathrm{))}\times{K}}{\sum_{{i}\mathrm{=1}}^{{N}}{{V}}^{\mathrm{2}}\mathrm{(}{i}\mathrm{)}}\times{100\%}$$

3 $$\:{SI}\mathrm{=}\frac{\sum_{{i}\mathrm{=1}}^{{N}}{{P}}^{\mathrm{2}}\mathrm{(}{i}\mathrm{)}-\sum_{{i}\mathrm{=1}}^{{N}}\mathrm{(}{P}\mathrm{(}{i}\mathrm{)}\times{V}\mathrm{(}{i}\mathrm{))/}{K}}{\sum_{{i}\mathrm{=1}}^{{N}}{{P}}^{\mathrm{2}}\mathrm{(}{i}\mathrm{)}}\times{100\%}$$

4 $$\:{K}\text{}\mathrm{=}\text{}\sqrt{\frac{{{V}}_{\mathrm{max}}}{{{P}}_{\mathrm{max}}}\times\frac{{{V}}_{{avg}}}{{{P}}_{{avg}}}}$$

where P(i) and V(i) are pixel values of perfusion and ventilation, N is the total number of lung pixels, Vmax and Pmax are the maximum pixel values, and Vavg and Pavg are the average pixel values. These three indices satisfy MI = (1 - DI) × (1 - SI).

5 $$\:{EIVQ}\mathrm{=}\frac{\sum_{{i}}{{a}}_{{i}}^{{V}}\times{RR}}{\text{}\sum_{{i}}{{a}}_{{i}}^{{P}}\times{HR}}$$

where $\:\left\{{{a}}_{{i}}^{{V}}\right\}$ is EIT ventilation image in one breath cycle (averaged over one minute), $\:\left\{{{a}}_{{i}}^{{P}}\right\}$ is EIT pulsatility image in one cardiac cycle (averaged over one minute), $\:{RR}$ is respiratory rate, and $\:{HR}$ is heart rate.

### Clinical data collection

Demographic data, including age, sex, and body mass index (BMI), along with laboratory parameters (D-dimer, troponin, NT-proBNP), were extracted from electronic medical records. Imaging data included CTPA and echocardiographic assessment of right ventricular (RV) function. RV dysfunction was defined by either an RV-to-left ventricular diameter ratio > 1.0 on CTPA or a tricuspid annular plane systolic excursion (TAPSE) < 16 mm on echocardiography [16].

### Statistical analysis

Statistical analyses were performed using SPSS 26.0 and Python 3.9. A two-sided *P* < 0.05 was considered significant. Continuous variables were tested for normality and presented as mean ± SD for normally distributed data and median (IQR) for non-normally distributed data. Categorical variables were reported as n (%). Between-group comparisons used the Mann–Whitney U test (two groups) and Kruskal–Wallis H test (multiple groups), with Dunn’s post-hoc test and Bonferroni correction for pairwise comparisons. Diagnostic performance was evaluated using ROC curve analysis, including AUC, 95% confidence interval, optimal cutoff value, sensitivity, specificity, PPV, and NPV. The optimal cutoff was determined using the Youden index, and PPV and NPV were calculated from the corresponding two-by-two contingency tables. DeLong’s test was used to compare AUC differences between models. A combined model of MI, DI, and SI was built using Z-score standardized logistic regression, with parameter contributions expressed as percentages of total absolute standardized coefficients.

## Results

### Baseline characteristics

A total of 106 participants were enrolled, including 53 PE patients and 53 healthy controls. Of the PE patients, 17 were recruited from Tsinghua Chang Gung Hospital and 36 from Chenzhou First People’s Hospital. As shown in Table 1, there were no significant differences in age (66.77 ± 14.36 vs. 62.55 ± 9.12 years; *P* = 0.074), gender distribution (67.9% male in the PE group vs. 60.4% in controls; *P* = 0.543), or body mass index (BMI, 24.00 ± 3.08 vs.24.74 ± 2.93 kg/m²; *P* = 0.207) between the two groups. However, PE patients had significantly higher median D-dimer levels compared to the control group (6.19 [IQR 3.61–13.53] vs. 0.30 [IQR 0.19–0.51] mg/L; *P* < 0.001).

**Table 1:** Baseline characteristics of the study population

Variables	Pulmonary embolism (*n* = 53)	Healthy controls (*n* = 53)	Statistic	*P*-value
Age, years (mean ± SD, 95%CI)	66.77 ± 14.36 (62.91–70.64)	62.55 ± 9.12 (60.09–65.00)	t = 1.81	0.074
Gender, female/male n (%)	17/36(32.1%/67.9%)	21/32(39.6%/60.4%)	χ²=0.37	0.543
BMI (kg/m², mean ± SD, 95%CI)	24.00 ± 3.08(23.17–24.83)	24.74 ± 2.93(23.95–25.53)	t = -1.271	0.207
D-dimer (mg/L, median, IQR)	6.19(3.61–13.53)	0.30(0.19–0.51)	Z = 8.676	< 0.001

Normally distributed data are presented as mean ± SD (95%CI), non-normally distributed data as median (IQR), and categorical data as n (%). Between-group comparisons were performed using the independent samples t-test, Mann-Whitney U test, or χ^2^ test. PE, pulmonary embolism; BMI, body mass index. Two-tailed P < 0.05 was considered statistically significant

### Pulsation parameters: PE patients vs. healthy controls

**Fig. 1:**
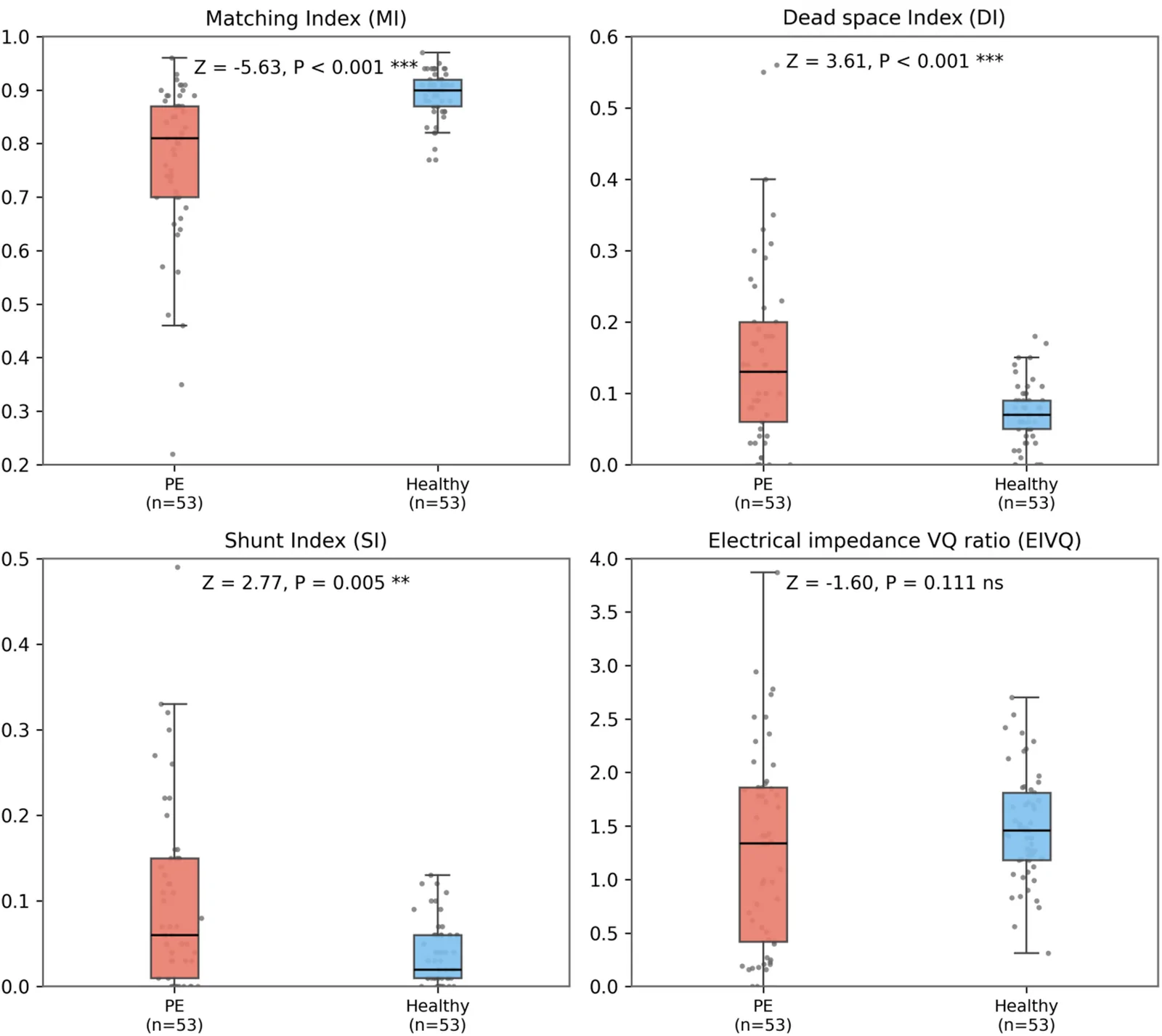
Comparison of EIT pulsation parameters between PE patients and healthy controls. Data presented as median (IQR). *P < 0.05, **P < 0.01, ***P < 0.001, ns = not significant (Mann-Whitney U test); EIT=electrical impedance tomography, PE=pulmonary embolism, MI=Matching Index, DI=Dead space index, SI=Shunt Index, EIVQ=Electrical Impedance VQ ratio

MI, DI, and SI, the three core pulsation parameters, differed significantly between the PE and control groups, with MI showing the strongest discriminative power. No significant difference was found for EIVQ (Table 2; Fig. 1). Specifically, MI was lower in the PE group (0.81 [ 0.70–0.87] vs.0.90 [0.87–0.92]; *P* < 0.001), while DI (0.13 [0.06–0.20] vs. 0.07 [0.05–0.09]; *P* < 0.001) and SI (0.06 [0.01–0.15] vs. 0.02 [0.01–0.06]; *P* = 0.005) were higher in the PE group.

**Table 2:** Comparisons of MI, DI, SI, and EIVQ between PE group and healthy control group

Parameter	PE (*n* = 53), median (IQR)	Healthy (*n* = 53), median (IQR)	Z value	*P* value
MI	0.81 (0.70–0.87)	0.90 (0.87–0.92)	-5.63	< 0.001
DI	0.13 (0.06–0.20)	0.07 (0.05–0.09)	3.61	< 0.001
SI	0.06 (0.01–0.15)	0.02 (0.01–0.06)	2.77	0.005
EIVQ	1.34 (0.42–1.86)	1.46(1.18–1.81)	-1.60	0.111

MI: Matching Index; DI: Dead space index; SI: Shunt Index; EIVQ: Electrical Impedance VQ ratio

### Pulsation parameters and PE risk stratification

**Fig. 2:**
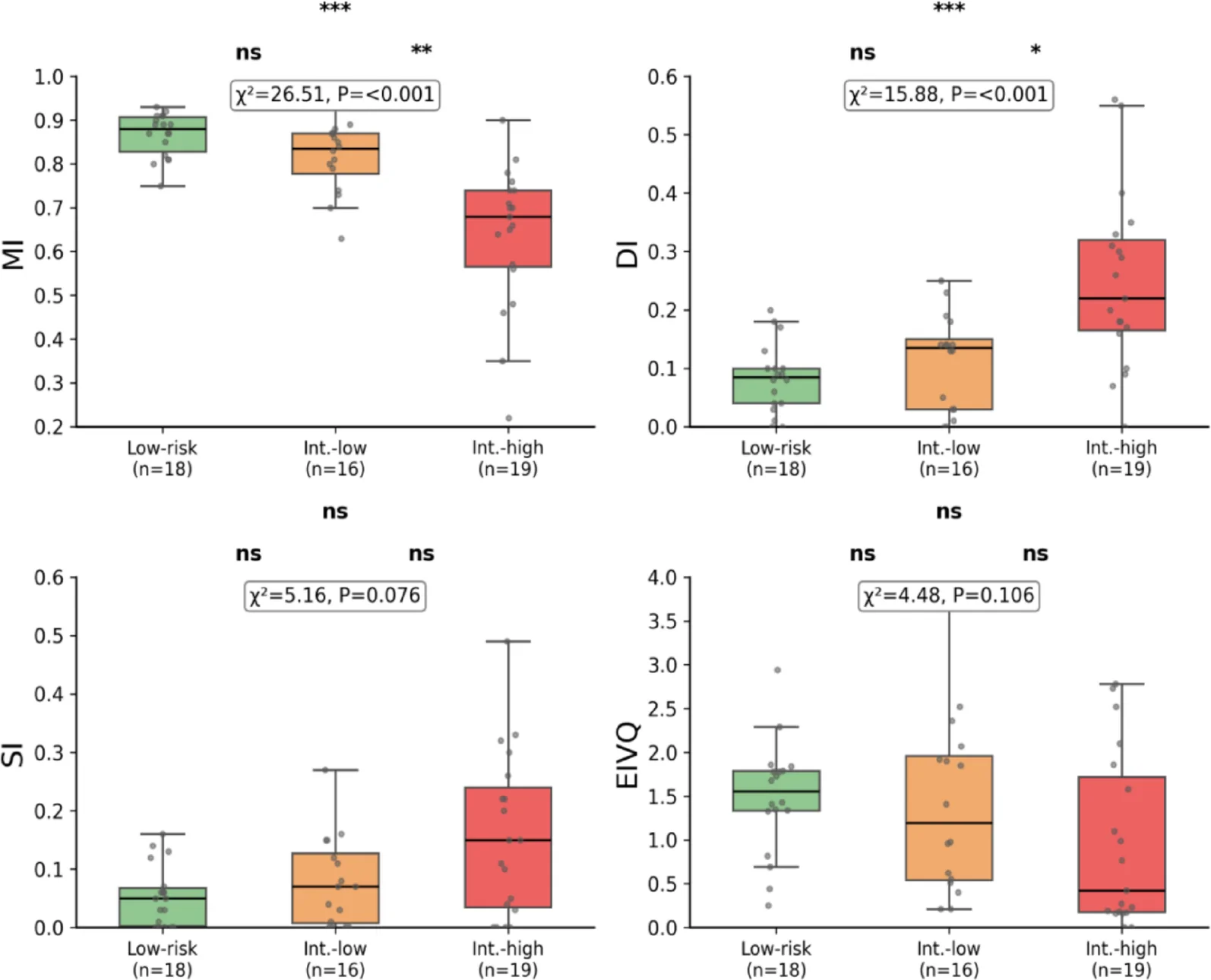
EIT pulsation parameters across PE risk stratification subgroups. Data presented as median (IQR). Dunn’s post-hoc test with Bonferroni correction showed the strongest differences between intermediate-high-risk and low-risk patients (both adjusted *P* < 0.001), with weaker but significant differences between intermediate-high-risk and intermediate-low-risk patients (MI: adjusted *P* < 0.01; DI: adjusted *P* < 0.05)

MI and DI showed significant overall differences across three PE risk stratification subgroups (MI: χ^2^=26.51, *P* < 0.001; DI: χ^2^=15.88, *P* < 0.001; Kruskal-Wallis test; Table 3; Fig. 2). Post-hoc analysis with Bonferroni correction (α = 0.017) confirmed lower MI and higher DI in intermediate-high-risk patients, with the strongest differences versus low-risk patients (both adjusted *P* < 0.001) and weaker differences versus intermediate-low-risk patients (MI: adjusted *P* < 0.01; DI: adjusted *P* < 0.05), while no significant differences were observed between low-risk and intermediate-low-risk subgroups (adjusted *P* > 0.05). SI and EIVQ exhibited no significant overall differences among the three subgroups (SI: χ^2^=5.16, *P* = 0.076; EIVQ: χ^2^=4.48, *P* = 0.106).

**Table 3:** EIT-related parameters across different risk stratification groups of pulmonary embolism

Parameter	Low-risk (*n* = 18)	Int.-low (*n* = 16)	Int.-high (*n* = 19)	Kruskal-wallis	Dunn’s post-hoc test with bonferroni correction
MI	0.88 (0.83–0.91)	0.83 (0.78–0.87)	0.68 (0.56–0.74)	χ²=26.51, *P* < 0.001	Low-risk vs. Int.-low: ns; Low-risk vs. Int.-high: ***; Int.-low vs. Int.-high: **
DI	0.08 (0.04–0.10)	0.14 (0.03–0.15)	0.22 (0.17–0.32)	χ²=15.88, *P* < 0.001	Low-risk vs. Int.-low: ns; Low-risk vs. Int.-high: ***; Int.-low vs. Int.-high: *
SI	0.05 (0.00–0.07)	0.07 (0.01–0.13)	0.15 (0.04–0.24)	χ²=5.16, *P* = 0.076	Low-risk vs. Int.-low: ns; Low-risk vs. Int.-high: ns; Int.-low vs. Int.-high: ns
EIVQ	1.55 (1.33–1.79)	1.19 (0.54–1.96)	0.42 (0.17–1.72)	χ²=4.48, *P* = 0.106	Low-risk vs. Int.-low: ns; Low-risk vs. Int.-high: ns; Int.-low vs. Int.-high: ns

Data are presented as median (interquartile range, IQR). Comparisons among groups were performed using Dunn’s post-hoc test with Bonferroni correction. Adjusted P values are indicated as follows: ***adjusted P < 0.001, **adjusted P < 0.01, *adjusted P < 0.05; ns, not significant. The Bonferroni-corrected significance threshold was 0.017

### Pulsation parameters and embolus location

**Fig. 3:**
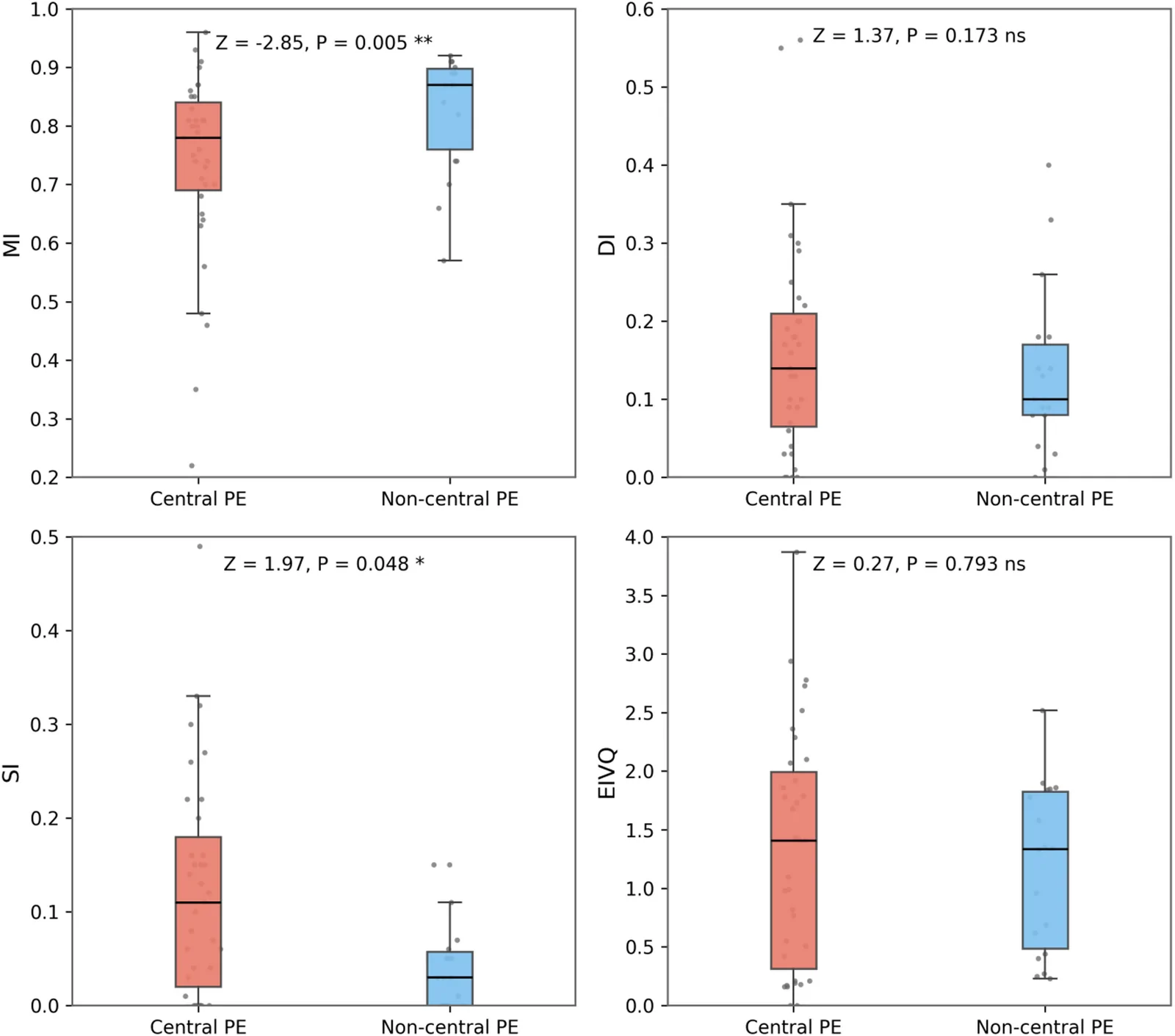
Comparison of EIT pulsation parameters between central PE (*n* = 35) and non-central PE (*n* = 18) patients. Data presented as median (IQR). *P < 0.05, **P < 0.01, ns = not significant (Mann-Whitney U test)

Central PE patients had significantly lower MI and higher SI than Non-central PE patients, with no significant differences in DI or EIVQ between the two subgroups (Table 4; Fig. 3). MI in Central PE patients was 0.76 [0.67–0.85], lower than that in Non-central PE patients (0.88 [0.81–0.89]; *P* < 0.05). In contrast, SI was higher in Central PE patients (0.11 [0.02–0.18] vs. 0.05 [0.00–0.07] in Non-central PE patients; *P* < 0.05).

**Table 4:** Comparison of EIT-related parameters between central and non-central PE groups

Parameter	Central PE (*n* = 35) (median, IQR)			Non-central PE (*n* = 18) (median, IQR)	Z value	*P* value
MI	0.76 (0.67–0.85)			0.88 (0.81–0.89)	-2.85	0.005
DI	0.16 (0.08–0.23)			0.10 (0.05–0.14)	1.37	0.173
SI	0.11 (0.02–0.18)			0.05 (0.00–0.07)	1.97	0.048
EIVQ	1.41 (0.32–1.99)			1.33 (0.48–1.78)	0.27	0.793

### Diagnostic efficacy of individual parameters and combined index

**Fig. 4:**
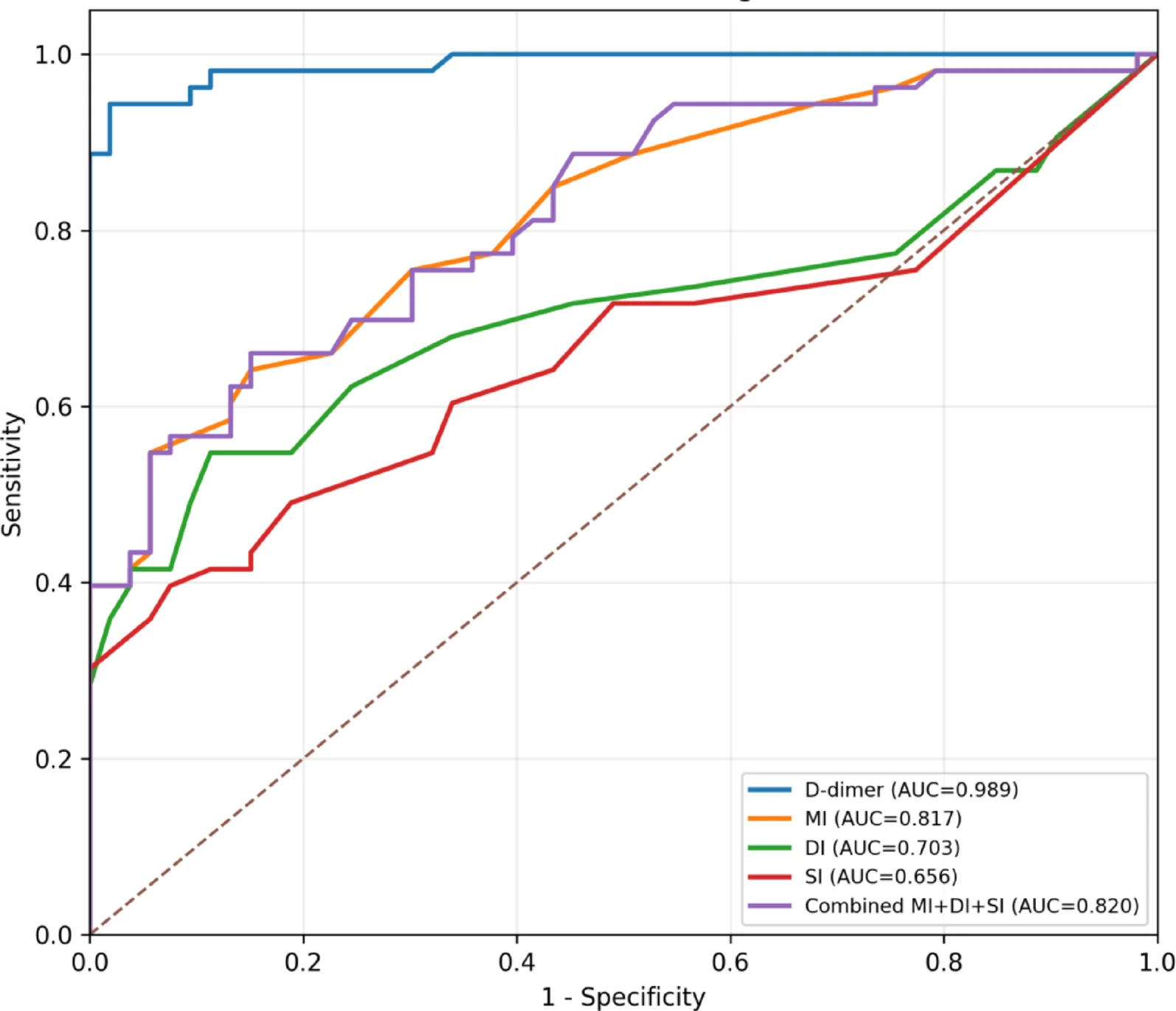
ROC curves of EIT parameters, combined model, and D-dimer for PE diagnosis. AUC values: D-dimer = 0.989, MI = 0.817, DI = 0.703, SI = 0.656, combined model = 0.820. DeLong’s test confirmed the combined model had a significantly higher AUC than DI and SI (both *P* < 0.05)

For comparison with the EIT-derived parameters, D-dimer was included in the ROC analysis. In this case-control cohort, D-dimer had an AUC of 0.989 (95% CI: 0.974–1.000). At the optimal cut-off value of ≥ 1.53 mg/L, the sensitivity, specificity, PPV, and NPV were 94.3%, 98.1%, 98.0%, and 94.5%, respectively.Among the EIT-derived parameters, MI had the highest AUC for PE diagnosis: 0.817 (95% CI: 0.737–0.897). Using the optimal cut-off value of ≤ 0.81, MI showed a sensitivity of 54.7%, specificity of 94.3%, PPV of 90.6%, and NPV of 67.6%. DI and SI had lower AUCs of 0.703 and 0.656, respectively. The combined MI + DI+SI model had an AUC of 0.820 (95% CI: 0.741–0.899), with a sensitivity of 66.0%, specificity of 84.9%, PPV of 81.4%, and NPV of 71.4%. The combined MI + DI+SI model showed only a marginal AUC improvement over MI alone, despite outperforming DI and SI (Table 5; Fig. 4).

**Table 5:** Diagnostic performance of EIT parameters for PE

Parameter	AUC (95% CI)	Cut-off value	Sensitivity (%)	Specificity (%)	PPV (%)	NPV (%)	Youden index
D-dimer	0.989 (0.974–1.000)	≥ 1.53	94.3	98.1	98.0	94.5	0.925
MI	0.817 (0.737–0.897)	≤ 0.81	54.7	94.3	90.6	67.6	0.491
DI	0.703 (0.599–0.808)	≥ 0.13	54.7	88.7	82.9	66.2	0.434
SI	0.656 (0.549–0.763)	≥ 0.11	39.6	92.5	84.0	60.5	0.321
Combined MI + DI+SI	0.820 (0.741–0.899)	≥ 0.48	66.0	84.9	81.4	71.4	0.509

AUC, area under the curve; CI, confidence interval

## Discussion

Pulmonary embolism (PE) remains a leading cause of morbidity and mortality in critically ill patients, and accurate diagnosis is crucial. Conventional diagnostic methods, including SPECT ventilation/perfusion (V/Q) imaging, clinical scoring systems with D-dimer thresholds, bedside ultrasound, and CTPA, have limitations in the ICU.These limitations include operator dependence, difficulties in assessing regional perfusion defects, and transport risks in unstable patients, especially for CTPA, which also involves radiation exposure and contrast-related nephropathy [17–23].

In this context, electrical impedance tomography (EIT) offers a promising non-invasive, contrast-free alternative for pulmonary perfusion assessment. Previous clinical studies have shown that EIT is effective in detecting regional perfusion defects and monitoring dynamic changes in PE, emphasizing its potential clinical utility [24–25]. Furthermore, international consensus supports EIT’s role in evaluating ventilation and perfusion in critically ill adults [26]. Preclinical studies have demonstrated that pulsatility-based signals in EIT reflect pulmonary blood flow, and three-dimensional EIT enhances spatial resolution and coverage [27–28]. Building on these advances, We applied a heart rate–adaptive frequency-domain band-pass filtering method to isolate cardiac-synchronous impedance changes while reducing respiratory and motion-related artifacts. The predefined 0.8–2.0 Hz range was used as the physiological cardiac-frequency range for heart rate-adaptive filtering, rather than to define pulmonary perfusion solely by frequency. In this method, the passband was centered on the estimated heart-rate frequency to isolate cardiac-synchronous impedance fluctuations. Conductance drift and respiratory-related impedance changes mainly occur at lower frequencies and are therefore attenuated by heart rate-adaptive band-pass filtering and subsequent averaging. Chest wall pulsation may contribute weak boundary-related signals, but these signals are spatially different from lung-field perfusion signals and can be reduced by lung ROI extraction and threshold-based image post-processing. Although cardiac bulk motion, extracardiac vascular oscillations, and large-vessel conduction artifacts may also occur at cardiac-synchronous frequencies, they are usually localized to the mediastinum, large-vessel regions, or adjacent cardiac areas. In contrast, pulmonary perfusion-related impedance changes are distributed within the lung fields. Therefore, the proposed method combines heart rate-adaptive temporal filtering with spatial ROI selection and image post-processing to enhance pulmonary perfusion-related pulsatility signals and reduce non-pulmonary cardiac artifacts.This approach enables real-time, bedside assessment of pulmonary perfusion without the need for breath-holding or contrast agents, offering a clinically actionable tool for monitoring PE in critically ill patients.

Our findings showed that PE patients exhibited significantly lower MI, and higher DI and SI compared to healthy controls, which aligns with the ventilation-perfusion mismatch characteristic of PE [29–30]. Specifically, the MI + DI+SI combined model demonstrated an AUC of 0.820 (95% CI: 0.741–0.899), confirming the diagnostic efficacy of this method and supporting its clinical relevance for PE detection. However, although MI, DI, and SI differed significantly between PE patients and healthy controls, overlap between groups was observed, suggesting that individual parameters may have limited value for per-patient diagnosis when used alone. The combined MI + DI+SI model provided more balanced diagnostic performance and should be considered a bedside adjunctive tool rather than a standalone diagnostic test.The limited incremental gain of the combined model suggests that MI may capture the dominant EIT-derived signal for distinguishing PE patients from healthy controls in this cohort.D-dimer showed the highest AUC in the present analysis, likely reflecting the marked difference in D-dimer levels between PE patients and healthy controls. However, EIT-derived pulsation indices provide bedside physiological information on regional pulmonary perfusion and ventilation-perfusion mismatch, which cannot be directly obtained from D-dimer testing. Therefore, the proposed EIT method should be viewed as a complementary bedside assessment tool rather than a replacement for established diagnostic strategies.The MI parameter exhibited the strongest discriminative power, consistent with its ability to reflect global perfusion disturbances in PE. The stepwise changes in MI and DI across PE risk strata, most evident in intermediate-high-risk patients, further support their potential utility for risk assessment.Our study also provides new insights into the relationship between embolus location and regional perfusion abnormalities. Central PE patients had significantly lower MI and higher SI compared to non-central PE patients. These findings suggest that EIT-derived indices can detect regional differences in pulmonary perfusion, which are not readily identified by traditional clinical evaluation methods. This supports the growing body of evidence that EIT can provide valuable information about embolus burden and location, potentially improving patient management.

The heart rate–adaptive frequency-domain band-pass filtering method improves the extraction of cardiac-synchronous impedance signals by centering the passband on the estimated heart-rate frequency. Since low-pass filtering mainly reflects ventilation-related impedance changes, a fixed 0.8–2.0 Hz band-pass filter was used as the non-adaptive reference method. However, this fixed range may include respiratory harmonics and cause ventilation-related contamination in the perfusion image. Compared with a fixed 0.8–2.0 Hz band-pass filter, the heart rate-adaptive method better preserved pulsatility signals and improved PE detection performance(see Additional file 1).The method allows non-invasive and contrast-free signal acquisition under free-breathing, spontaneous-breathing, and mechanically ventilated conditions. It does not require saline injection, central venous access, or breath-holding.Respiratory activity, conductance drift, and slow movement-related changes mainly occur at lower frequencies and can be attenuated by band-pass filtering and sliding-average processing. Electrode noise outside the passband can also be reduced. Therefore, EIT pulsatility imaging may serve as a real-time bedside tool for monitoring PE-related pulmonary perfusion abnormalities in critically ill patients.However, this method has several limitations. It is most suitable for patients with heart-rate frequencies within the predefined cardiac range of 0.8–2.0 Hz, corresponding to approximately 48–120 beats/min. In severe bradycardia, extreme tachycardia, atrial fibrillation, hypotension, or low-perfusion states, the pulsatility signal may become weak or unstable. This may reduce the reliability of heart-rate identification and perfusion assessment. Abrupt movement, unstable electrode contact, or in-band noise may also affect signal quality. In these cases, manual adjustment of the filtering bounds or further algorithm optimization may be needed.

Several limitations should be considered. This was a retrospective case-control study, and the control group consisted of healthy volunteers rather than patients with suspected PE and negative CTPA findings.Therefore, these diagnostic estimates, including those of D-dimer and EIT-derived parameters, should be interpreted cautiously, as the use of healthy controls may overestimate diagnostic performance compared with a real-world suspected-PE population.The EIT pulsatility method provides an indirect assessment of pulmonary perfusion based on cardiac-synchronous impedance changes. Although filtering and lung-region ROI extraction were applied, the signal may still be affected by limited spatial resolution, electrode contact, body movement, cardiac-related artifacts, arrhythmia, or low-perfusion states.Prospective studies enrolling consecutive patients with suspected PE are needed to validate these findings and to compare EIT-derived parameters with established diagnostic strategies.

In conclusion, the heart rate–adaptive frequency-domain band-pass filtering method for EIT provides a rapid, non-invasive bedside approach for assessing pulmonary perfusion-related abnormalities in patients with PE. It offers physiological information that may complement conventional diagnostic methods and support PE risk stratification. Future studies should validate this real-time EIT approach in prospective suspected-PE cohorts and further define its role in bedside PE diagnosis and risk stratification.

## Supplementary Information

Below is the link to the electronic supplementary material.

Supplementary Material 1: Quantitative comparison of heart rate–adaptive versus fixed frequency filtering for EIT pulsation signal extraction.

## Data Availability

Data supporting the findings are available from corresponding authors upon reasonable request.

## References

[1] Konstantinides SV, Meyer G, Becattini C, Bueno H, Geersing GJ, Harjola VP, et al. 2019 ESC Guidelines for the diagnosis and management of acute pulmonary embolism developed in collaboration with the European Respiratory Society (ERS). Eur Heart J. 2020;41:543–603. 10.1093/eurheartj/ehz405. 31504429

[2] Chopard R, Behr J, Vidoni C, Ecarnot F, Meneveau N. An update on the management of acute high-risk pulmonary embolism. J Clin Med. 2022;11(16):4807. 10.3390/jcm11164807. PMC9409943 36013046

[3] de Wit K, D’Arsigny CL. Risk stratification of acute pulmonary embolism. J Thromb Haemost. 2023;21(11):3016–23. 10.1016/j.jtha.2023.05.003. 37187357

[4] Zantonelli G, Cozzi D, Bindi A, Cavigli E, Moroni C, Luvarà S, Grazzini G, Danti G, Granata V, Miele V. Acute Pulmonary Embolism: Prognostic Role of Computed Tomography Pulmonary Angiography (CTPA). Tomography. 2022;8(1):529–39. 10.3390/tomography8010042. PMC8880178 35202207

[5] Diaz-Lorenzo I, Alonso-Burgos A, Friera Reyes A, et al. Current role of CT pulmonary angiography in pulmonary embolism: A state-of-the-art review. J Imaging. 2024;10(12):323. 10.3390/jimaging10120323. PMC11678867 39728220

[6] Fan BE, Lippi G, Favaloro EJ. D-dimer levels for the exclusion of pulmonary embolism: making sense of international guideline recommendations. J Thromb Haemost. 2024;22:604–8. 10.1016/j.jtha.2023.12.015. 38135252

[7] Te Haara SR, De Rezende H, Wang C. Diagnostic test accuracy of the YEARS algorithm for pulmonary embolism: a systematic review and meta-analysis. Sultan Qaboos Univ Med J. 2024;24:491–500. 10.18295/squmj.1.2024.007. PMC11614012 39634805

[8] Stals MAM, Beenen LFM, Coppens M, Faber LM, Hofstee HMA, Hovens MMC, et al. Performance of the 4-Level Pulmonary Embolism Clinical Probability Score (4PEPS) in the diagnostic management of pulmonary embolism: an external validation study. Thromb Res. 2023;231:65–75. 10.1016/j.thromres.2023.09.010. 37816274

[9] Scaramuzzo G, Pavlovsky B, Adler A, Baccinelli W, Bodor DL, Damiani LF, et al. Electrical impedance tomography monitoring in adult ICU patients: state-of-the-art, recommendations for standardized acquisition, processing, and clinical use, and future directions. Crit Care. 2024;28:377. 10.1186/s13054-024-05173-x. PMC11577873 39563476

[10] Xu J, Wang L, Tong L, Li H, Wu X, Wang Y, et al. Novel three-dimensional electrical impedance tomography for noninvasive detection of lung perfusion. J Thorac Dis. 2025;17:4837–48. 10.21037/jtd-2025-275. PMC12340291 40809230

[11] Xu M, He H, Long Y. Lung Perfusion Assessment by Bedside Electrical Impedance Tomography in Critically Ill Patients. Front Physiol. 2021;12:748724. 10.3389/fphys.2021.748724. PMC8548642 34721072

[12] He H, Long Y, Frerichs I, Zhao Z. Detection of Acute Pulmonary Embolism by Electrical Impedance Tomography and Saline Bolus Injection. Am J Respir Crit Care Med. 2020;202(6):881–2. 10.1164/rccm.202003-0554IM. 32469613

[13] Gao Y, Cai Q, Yuan S, Xu M, Wu S, Adler A, Long Y, Zhao Z, He H. Lung perfusion estimation by saline-contrast EIT without breath hold: a randomized cross-over trial. Crit Care. 2026;30(1):55. 10.1186/s13054-025-05823-8. PMC12869906 41486260

[14] Grant CA, Pham T, Hough J, Riedel T, Stocker C, Schibler A. Measurement of ventilation and cardiac related impedance changes with electrical impedance tomography. Crit Care. 2011;15:R37. 10.1186/cc9985. PMC3222074 21266025

[15] Zadehkoochak M, Blott BH, Hames TK, George RF. Pulmonary perfusion and ventricular ejection imaging by frequency domain filtering of EIT (electrical impedance tomography) images. Clin Phys Physiol Meas. 1992;13(Suppl A):191–6. 10.1088/0143-0815/13/a/037. 1587100

[16] Pruszczyk P, Kurnicka K, Ciurzyński M, Hobohm L, Thielmann A, Sobkowicz B, et al. Defining right ventricular dysfunction by echocardiography in normotensive patients with pulmonary embolism. Pol Arch Intern Med. 2020;130:741–7. 10.20452/pamw.15459. 32579314

[17] Thomas SE, Weinberg I, Schainfeld RM, Rosenfield K, Parmar GM. Diagnosis of Pulmonary Embolism: A Review of Evidence-Based Approaches. J Clin Med. 2024;13(13):3722. 10.3390/jcm13133722. PMC11242034 38999289

[18] Oh JK, Park JH. Role of echocardiography in acute pulmonary embolism. Korean J Intern Med. 2023;38:456–70. 10.3904/kjim.2022.273. PMC10338244 36587934

[19] Du Y, Yang A, Wang X. Accuracy of transthoracic lung ultrasound for diagnosing pulmonary embolism: An updated systematic review and meta-analysis. Thromb Res. 2024;241:109112. 10.1016/j.thromres.2024.109112. 39126978

[20] Melo RH, Gioli-Pereira L, Lourenço ID, Da Hora Passos R, Bernardo AT, Volpicelli G. Diagnostic accuracy of multi-organ point-of-care ultrasound for pulmonary embolism in critically ill patients: a systematic review and meta-analysis. Crit Care. 2025;29(1):162. 10.1186/s13054-025-05359-x. PMC12020239 40269937

[21] Iftikhar IH, Iftikhar NH, Naeem M, BaHammam A. SPECT Ventilation/Perfusion Imaging for Acute Pulmonary Embolism: Meta-analysis of Diagnostic Test Accuracy. Acad Radiol. 2024;31(2):706–17. 10.1016/j.acra.2023.06.024. 37487880

[22] Righini M, Robert-Ebadi H, Le Gal G. Age-Adjusted and Clinical Probability Adapted D-Dimer Cutoffs to Rule Out Pulmonary Embolism: A Narrative Review of Clinical Trials. J Clin Med. 2024;13(12):3441. 10.3390/jcm13123441. PMC11204230 38929970

[23] Duffy J, Berger FH, Cheng I, Shelton D, Galanaud JP, Selby R, Laing K, Fedorovsky T, Matelski J, Hall J. Implementation of the YEARS algorithm to optimise pulmonary embolism diagnostic workup in the emergency department. BMJ Open Qual. 2023;12(2):e002119. 10.1136/bmjoq-2022-002119. PMC10231008 37217241

[24] Magaña Bru I, Delgado Arroyo A, Suarez Sipmann F. Electrical impedance tomography for the detection and management optimization of pulmonary embolism. Med Intensiva (Engl Ed). 2025;49(7):502134. 10.1016/j.medine.2025.502134. 39818528

[25] Wang Q, He H, Yuan S, Jiang J, Chi Y, Long Y, Zhao Z. Early bedside detection of pulmonary perfusion defect by electrical impedance tomography after pulmonary endarterectomy. Pulm Circ. 2024;14(2):e12372. 10.1002/pul2.12372. PMC11063724 38699668

[26] He H, Zhao Z, Becher T, Bellani G, Yoshida T, Amato MBP, et al. Recommendations for lung ventilation and perfusion assessment with chest electrical impedance tomography in critically ill adult patients: an international evidence-based and expert Delphi consensus study. EClinicalMedicine. 2025;89:103575. 10.1016/j.eclinm.2025.103575. PMC12555788 41158153

[27] Li J, Zhu M, Guo Y, Li W, He Q, Wang Y, et al. Dynamic EIT technology for real-time non-invasive monitoring of acute pulmonary embolism: a porcine model experiment. Respir Res. 2025;26:7. 10.1186/s12931-024-03090-9. PMC11715541 39780173

[28] Gao Y, Zhang K, Li M, Yuan S, Wang Q, Chi Y, et al. Feasibility of 3D-EIT in identifying lung perfusion defect and V/Q mismatch in a patient with VA-ECMO. Crit Care. 2024;28:90. 10.1186/s13054-024-04865-8. PMC10956177 38509551

[29] Deng M, Li N, Wang J, Zhao S, Yu M. Bedside detection and monitoring of pulmonary embolism using electrical impedance tomography. Front Physiol. 2026;17:1729553. 10.3389/fphys.2025.1729553. PMC12958555 41788880

[30] He H, Chi Y, Long Y, Yuan S, Zhang R, Frerichs I, Möller K, Fu F, Zhao Z. Bedside Evaluation of Pulmonary Embolism by Saline Contrast Electrical Impedance Tomography Method: A Prospective Observational Study. Am J Respir Crit Care Med. 2020;202(10):1464–8. 10.1164/rccm.202005-1780LE. PMC7667910 32585116

