# Supplementary material for: Frequency-domain band-pass filtering enhanced pulsation method for pulmonary embolism diagnosis and risk stratification: a two-center retrospective study
Source: Crit Care. 2026 Jul 10;30:370. doi: 10.1186/s13054-026-06173-9 (PMC13371360; doi:10.1186/s13054-026-06173-9)
Supplement: Supplementary file 1 — Supplementary Material 1: Quantitative comparison of heart rate–adaptive versus fixed frequency filtering for EIT pulsation signal extraction. [file 13054_2026_6173_MOESM1_ESM.pdf]

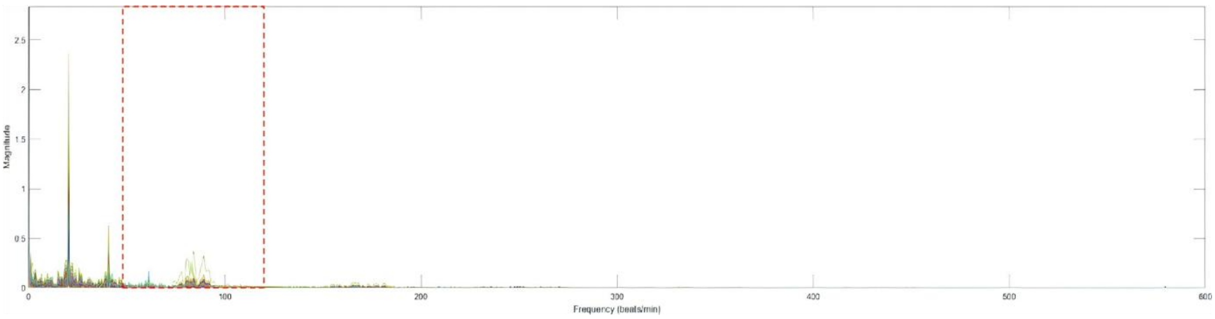

Figure A. Frequency spectrum of the EIT signal. The red dashed box indicates the fixed band-pass filtering range. In this representative case, the range includes the third harmonic of respiration.

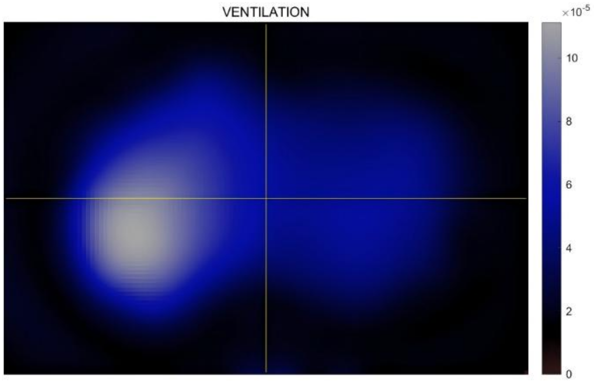

Figure B Ventilation image of a patient with pulmonary embolism.

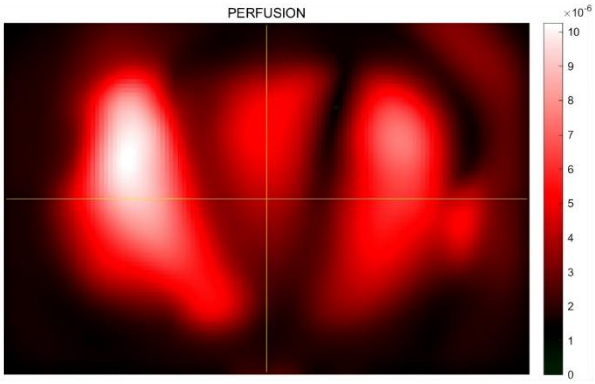

Figure C Perfusion image obtained using the proposed heart rate-adaptive filtering method.

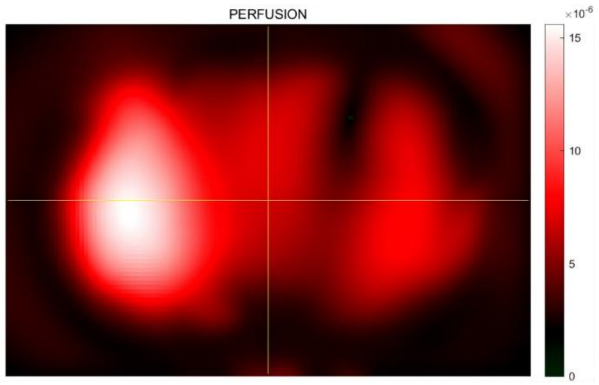

Figure D. Perfusion image obtained using fixed band-pass filtering. The spatial pattern becomes more similar to the ventilation image, indicating contamination by respiratory-related components.

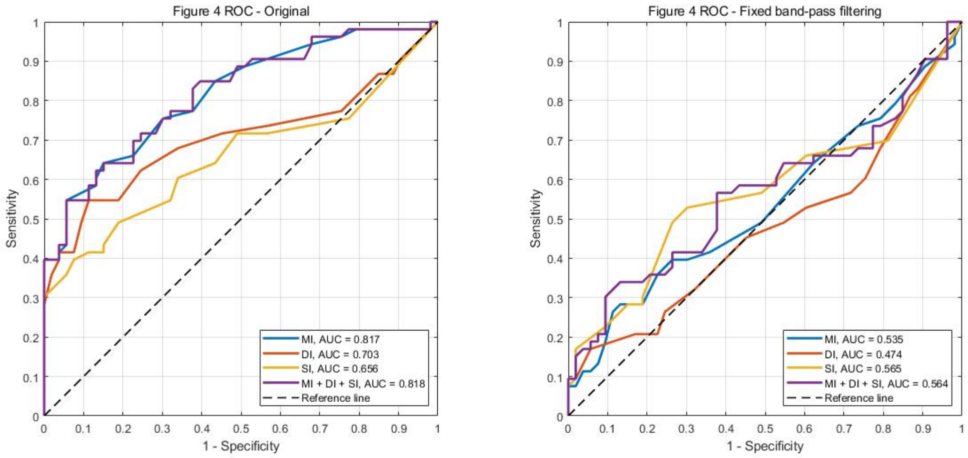

Figure E. Comparison of pulmonary embolism detection performance between the proposed heart rate-adaptive filtering method and fixed band-pass filtering. ROC curves were generated using MI, DI, SI, and the combined MI + DI + SI model.
